# Intermittency and Predictability of a Cafeteria Diet Shape Food Intake, Adiposity, and Neurobehavioral Outcomes in Rats

**DOI:** 10.3390/nu18121913

**Published:** 2026-06-12

**Authors:** Rebeca Vindas-Smith, Andrey Sequeira-Cordero, Maripaz Castro, Juan C. Brenes

**Affiliations:** 1 Instituto de Investigaciones en Salud, Universidad de Costa Rica, San José 11501-2060, Costa Rica; rebeca.vindas@ucr.ac.cr (R.V.-S.); andrey.sequeiracordero@ucr.ac.cr (A.S.-C.); 2 Programa de Doctorado en Ciencias, Sistema de Estudios de Posgrado, Universidad de Costa Rica, San José 11501-2060, Costa Rica; 3 Centro de Investigación en Neurociencias, Universidad de Costa Rica, San José 11501-2060, Costa Rica; 4 Laboratorio de Ensayos Biológicos, Universidad de Costa Rica, San José 11501-2060, Costa Rica; maripaz.castro@ucr.ac.cr; 5 Instituto de Investigaciones Psicológicas, Universidad de Costa Rica, San José 11501-2060, Costa Rica

**Keywords:** anxiety, dietary habits, feeding schedule, junk food, neuroplasticity, obesity, overeating, reward, palatability, ultra-processed foods

## Abstract

**Background/Objective**: Highly palatable foods are pleasurable and motivational stimuli that activate the brain’s reward system and can induce overeating in the absence of physiological needs. This study investigated how different access patterns to a cafeteria diet influence food intake, body weight-related parameters, and metabolic and neurobehavioral outcomes. **Methods**: At postnatal day 31, forty male Wistar rats were assigned to a standard diet or a cafeteria diet with continuous, predictable intermittent, or unpredictable intermittent access. After 10 weeks, the open-field and sucrose-preference tests assessed exploratory and anxiety-like behaviors and reward-related responses, respectively. Body composition, serum biochemical parameters, neurotransmitter content, and mRNA and protein levels were analyzed in reward-related brain regions. **Results**: Intermittent access increased food intake on cafeteria days compared with continuous access, with unpredictable access yielding the highest intake. Continuous-access rats exhibited higher final body weight and fat accumulation than chow-fed Control rats. Despite similar body weight, both intermittent-access groups had higher visceral adiposity, obesity indices, and adverse metabolic outcomes than the Control group. All cafeteria-fed rats displayed anxiety-like behavior, and all groups preferred sucrose except the continuous-access group. Molecular analyses revealed region-specific differences in gene expression related to neuroplasticity, stress response, and epigenetic regulation that varied with access pattern and predictability. **Conclusions**: Our results suggest that, beyond diet composition, the pattern and predictability of food access are key determinants of feeding behavior. Intermittent access increases the motivational value of the cafeteria diet, promoting overeating and driving reward- and stress-related neuroadaptations with potential metabolic and mental health implications.

## 1. Introduction

Today’s food environments are characterized by the widespread availability and affordability of calorie-dense, highly palatable (HPFs) and ultra-processed foods (UPFs), which are made with combinations of sugar, fat, and sodium at levels rarely found in natural foods [1]. Excessive consumption of these foods, and exposure to the cues associated with them—often perceived as highly pleasurable stimuli—can drive hedonic and motivational eating by activating the brain’s reward system, leading to the release of dopamine in regions involved in reward processing, learning, and executive functions, such as the nucleus accumbens (NAc), the dorsal striatum (DS), the hippocampus (HPC), and the medial prefrontal cortex (mPFC) [2].

Adolescence is a critical stage of development during which there is an increased tendency to engage in rewarding and often risky behaviors [3], such as overeating HPFs. These behavioral patterns could persist into adulthood and increase the risk of adverse metabolic and mental health outcomes associated with poor diet quality [4,5]. In Westernized societies, the consumption of HPFs and UPFs has become very common among adolescents and represents a significant risk factor for rising obesity rates in this population [6].

Data from preclinical and human studies suggest that increased HPF consumption is influenced not only by macronutrient composition and energy density but also by patterns of food availability [7,8,9,10,11,12,13,14]. In real-world settings, people often alternate between healthier and less healthy eating habits, including occasional excessive consumption of preferred foods. This pattern has been observed in behaviors such as yo-yo dieting, emotional eating, “cheat meals or days,” and stress-induced eating, as well as in certain clinical conditions (e.g., binge eating disorder) [15,16,17]. These patterns underscore the importance of studying intermittent access to palatable foods in animal models to investigate how fluctuations in food availability influence reward-related behaviors, stress responses, and long-lasting neurobiological effects.

In rodents, intermittent access to palatable foods (e.g., limited to specific times or days) induces binge-like phenotypes and behavioral sensitization, characterized by an intensified behavioral response after repeated exposure, which parallels mechanisms observed with intermittent access to drugs of abuse [8,9,12,18,19,20,21]. However, a less explored factor is food access under unpredictable conditions [10,22,23]. Uncertainty about food availability in natural environments invigorates food-seeking behaviors in mammals and birds, leading to increased food intake and body fat reserves compared with predictable conditions [24]. These behaviors involve incentive-motivational processes that subserve survival. Similar mechanisms likely operated during human evolution but have become maladaptive in modern obesogenic environments [24].

Therefore, the present study aimed to examine three patterns of access to a cafeteria-style diet in male Wistar rats to investigate how continuous versus intermittent access, as well as the predictability of intermittent feeding schedules (i.e., predictable vs. unpredictable), differentially affect food intake, reward-related behaviors, psychomotor responses, and metabolic and neurobiological outcomes. The cafeteria (CAF) diet is a voluntary-choice model that mirrors dietary patterns observed in Westernized societies. It provides animals with HPFs similar to those commonly consumed by humans and is well-suited for investigating neurobiological adaptations to palatable diets [25]. Animals were exposed to the CAF diet throughout adolescence and young adulthood to improve the translational relevance of the model, as this period marks the onset of obesity, and to evaluate neurobehavioral consequences associated with early exposure to HPFs and UPFs. Therefore, we analyzed the expression of genes involved in neuroplasticity (brain-derived neurotrophic factor (*Bdnf*), neurotrophic receptor tyrosine kinase 2 (*Ntrk2*), and cAMP response element-binding protein (*Creb1*)), stress response (corticotropin-releasing factor (*Crf*), and epigenetic modifications (DNA methyltransferase 3 alpha (*Dnmt3a*) and histone deacetylase 5 (*Hdac5*)) to assess their role in motivated behavior in the NAc, DS, mPFC, and HPC—key brain regions for motivation, reward, and cognition [26,27,28,29]. This multilevel approach could provide insights into the importance of considering not only dietary composition but also the temporal dynamics of food access in the development of obesity and related disorders.

We hypothesized that exposure to a highly palatable diet during adolescence and early adulthood would alter food intake, food preferences, and body weight-related parameters, accompanied by changes in the expression of genes involved in neuroplasticity. In addition, we hypothesized that different patterns of CAF diet exposure would differentially modulate these metabolic and neurobehavioral effects.

## 2. Materials and Methods

This study was approved by the Institutional Committee for Animal Care and Use of the Universidad de Costa Rica (CICUA-022-19), and all experimental procedures complied with the National Research Council’s Guide for the Care and Use of Laboratory Animals.

### 2.1. Animals and Experimental Design

**Figure 1:**
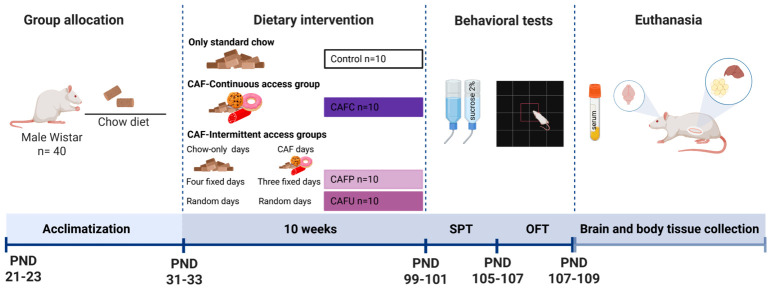
Experimental timeline. At PND 31 ± 2, rats were single-housed and assigned to one of four groups: Control, CAFP, CAFU, or CAFC. The Control group was fed the standard diet throughout the experimental protocol, while the CAFC group had continuous access to the CAF diet. The CAFP group had access to the CAF diet on Monday, Wednesday, and Friday, and received the standard diet on chow-only days. The CAFU group had access to the CAF diet on randomly assigned days each week and received the standard diet on chow-only days. All animals were exposed to the dietary intervention until PND 107 ± 2; however, food intake and body weight were recorded for 10 weeks. From PND 99 to 106 ± 2, two SPTs and two OFTs were conducted for behavioral assessment. Before euthanasia, final body weight and body length were measured. At PND 107 ± 2, trunk blood was collected for serum biochemical analyses, and the liver and body fat depots were dissected and weighed. Finally, brain regions involved in reward-related behaviors were isolated to quantify neurotransmitter content and mRNA and protein levels of genes involved in diet-induced neuroplasticity. PND: postnatal day; CAF: cafeteria diet; CAFP: cafeteria predictable intermittent-access group; CAFU: cafeteria unpredictable intermittent-access group; CAFC: cafeteria continuous-access group; SPT: sucrose preference test; OFT: open field test. Vindas-Smith, R. (2026) https://BioRender.com/y76vgf9.

Forty male Wistar rats (HsdBrlHan: WIST) obtained from Laboratorio de Ensayos Biológicos (LEBi^®^) of the Universidad de Costa Rica, San José, Costa Rica, were transported to the animal facility on postnatal day (PND) 21 ± 2 and housed in littermate groups in polycarbonate cages (59 cm × 39 cm × 20 cm) with wood-shaving bedding. Animals were maintained in a climate-controlled room at 22 ± 2 °C and 56–64% relative humidity, with a 12:12 h light/dark cycle (lights on at 07:00) and 15–20 air changes per hour. All rats had free access to a standard laboratory rodent diet (LabDiet 5010, LabDiet, Richmond, IN, USA) and water. Cage cleaning and bedding replacement were performed twice weekly. A 10-day acclimatization period was provided prior to allocation to the experimental groups (Figure 1). The sample size estimate was based on previous studies conducted in our laboratory that reported robust effect sizes for food intake and body fat accumulation [27,30,31].

Animals were single-housed and randomly assigned using a computer-generated randomization sequence to four groups (*n* = 10 rats per group), accounting for variations in body weight, food and water intake, and dam of origin, as previously reported [27,31]. Groups differed in their exposure to the CAF diet (Figure 1). **Control group:** received the standard chow diet exclusively throughout the experimental period (LabDiet 5010, 3.4 kcal/g). The standard diet provided 58% kcal from carbohydrates, 29% from protein, and 13% from fat. According to their feeding schedule, intermittent-access groups had access to the CAF diet on some days (CAF days) and exclusively to the standard diet on other days (chow-only days), as follows: **Cafeteria predictable intermittent-access group (CAFP):** had ~24 h access to the CAF diet on fixed days each week (CAF days: Monday, Wednesday, and Friday) and received only standard chow on the remaining days (chow-only days). **Cafeteria unpredictable intermittent-access group (CAFU):** received ~24 h access to the CAF diet on randomly assigned days each week (CAF days) and only the standard diet on the remaining days. To minimize anticipatory behavior, CAF food availability was randomized within a 9-day block. Food intake was recorded weekly to maintain consistency with the other groups. Access to the CAF diet ranged from 2 to 5 days per week, randomly selected. **Cafeteria continuous-access group (CAFC):** had exclusive access to the CAF diet throughout the experimental period. The CAF diet consisted of daily combinations of three palatable food items provided together with standard chow pellets, and the energy density varied across days. These combinations were prepared using 17 food items (16 commercial products plus the standard diet), whose nutritional information and energy density, as well as frequency of inclusion, are presented in Tables S1 and S2, respectively. On average, the CAF diet provided 42% kcal from carbohydrates, 13% from protein, and 45% from fat [27,31].

Each three-food combination consisted of either two savory and one sweet item or vice versa. All food items, including standard pellets for all groups, were changed daily between 15:00 and 17:00 h. During CAF days, intermittent-access groups (CAFP and CAFU) received the same food combinations as the CAFC group. Based on our previous evidence that nine weeks are sufficient to alter body composition [31], an 11-week protocol was implemented to ensure robust biometric changes; however, food intake was recorded only through week 10. Rats remained on their assigned diets until euthanasia. The CAFP and CAFU groups were exposed to the CAF diet for 31 days according to their respective schedules, whereas the CAFC group was exposed for 68 days. To reduce potential distress associated with individual housing, rats were allowed to interact with their corresponding group partners for at least two hours, three times per week [31], as social interaction is an important welfare consideration in rats. Cage positions within the rack were rotated twice weekly during cage cleaning and bedding replacement to minimize potential environmental confounders associated with housing location.

At week 11, two sucrose preference tests (SPTs) and two open field tests (OFTs) were conducted. Finally, 24 h after the last OFT, the animals were euthanized by decapitation for tissue processing (Figure 1). The experimental protocol extended from early adolescence to adulthood. All procedures were counterbalanced across experimental groups to minimize potential procedural confounders and ensure representation of all groups throughout the processing period. Serum, neurochemical, and expression analyses were performed by investigators blinded to group allocation. Predefined exclusion criteria were established a priori in the approved experimental protocol. Animals were to be excluded if they exhibited significant body weight loss relative to baseline values, abnormal behavior, or lethargy indicative of compromised health or welfare. No animals met these exclusion criteria, and no adverse events, expected or unexpected, occurred during the study. All rats were monitored six times per week during body weight measurements to assess body weight changes and general welfare indicators, including overall appearance, excessive grooming, posture, and locomotor activity. No animals showed signs requiring early removal from the study or humane euthanasia.

### 2.2. Food Intake and Body Weight Measures

Each food item was weighed individually before and after being offered to the animals, including any leftovers recovered from the cages. Food intake (g) was calculated as the difference between initial and final weights, adjusted for potential changes in food mass due to hydration or dehydration. The correction factor was obtained as previously described [31]. Briefly, an identical food combination was placed in an animal-free polycarbonate cage with bedding for this purpose. Using the corrected consumption values, energy (kcal) and macronutrient intake (g) were determined from the nutritional information on product labels. Food intake and body weight were measured six times per week. 

Daily food and energy intake values were recorded as absolute values and normalized to each animal’s body weight (kg) measured on the same corresponding day. Weekly mean daily values per animal were then calculated for food intake, energy intake, and body weight, and overall mean values across weeks were subsequently obtained. Additional analyses of food intake behavior were conducted separately for chow-only and CAF days. During CAF days, food intake was compared between CAFC and intermittent-access groups, whereas during chow-only days, comparisons included the Control and intermittent-access groups to evaluate the effects of intermittent access. The CAFP and CAFU groups were also compared under both conditions to assess the effects of predictability. Weekly mean daily intake per animal and overall mean values across weeks were calculated for each group based on the number of weekly exposure days to the chow or CAF diet, and CAF diet composition was evened out between these groups to the largest extent possible. To account for developmental effects, food intake was additionally analyzed in two stages: weeks 1–5 (adolescence) and weeks 6–10 (early adulthood), allowing assessment of age-dependent changes across the experimental period. 

At the end of the experiment, animals were weighed and their naso-anal length was measured to compute the body mass index (BMI) [final body weight (g)/body length (cm^2^)] and the Lee index [cube root of final body weight (g)/body length (cm)] × 1000, according to Novelli et al. (2007) [32]. Visceral fat depots were dissected and weighed to assess body fat accumulation. The percentage of total white adipose tissue (WAT) was calculated as [(perigonadal fat (g) + intra-abdominal fat (g))/final body weight (g)] × 100. The liver was also removed and weighed. Three investigators, responsible for diet administration according to the experimental schedule and for food intake and body weight measurements, were aware of group allocation, as blinding was not feasible because the diets were identifiable. Nonetheless, all measurements were collected and handled as uniformly as possible across all experimental groups to minimize procedural bias.

### 2.3. Behavioral Tests

#### 2.3.1. Sucrose Preference Test

Two SPTs were assessed using a two-bottle choice paradigm to evaluate reward-related behavior. The first test was conducted over a 48 h period on PND 99 ± 2. Each rat was provided with two identical bottles, one containing 400 mL of a 2% sucrose solution and the other containing 400 mL of water. Although this phase was intended primarily to promote habituation—especially for Control rats with no previous exposure to sweet substances—it was also included in the analysis. To reduce potential side bias, the positions of the two bottles were interchanged after 24 h. Consumption during this 48 h period was quantified to characterize initial sucrose responsiveness across experimental groups. 

The second SPT was conducted 72 h after the start of the first assessment. During this 24 h test, animals received 250 mL of the 2% sucrose solution and 250 mL of water. Water and sucrose consumption were calculated using a correction factor to account for fluid loss unrelated to drinking behavior. For this purpose, an additional empty cage equipped with the same bottles and solutions used for the animals was included in parallel during each testing period. The volume loss observed in this control cage was used to estimate dripping losses and was subtracted from each animal’s liquid consumption. Consumption of water and sucrose, and total fluid intake (mL) were measured and normalized by body weight (kg). Sucrose preference was calculated as follows: [sucrose intake (mL)/(sucrose + water intake) (mL)] × 100. All animals had ad libitum access to standard laboratory chow only during both tests to avoid confounding interactions with the palatable CAF diet. The sucrose solution was freshly prepared by dissolving sugar (99.5% purity) in tap water.

#### 2.3.2. Open Field Test

The two OFTs were performed on PND 105 ± 2 over two consecutive days, in accordance with previously published protocols [27,33], to assess spontaneous locomotor and exploratory activity in a novel environment. The order of testing was counterbalanced across experimental groups. For the second testing session, the order was additionally counterbalanced according to both the experimental group and the timing of testing relative to the first session. Each animal was individually introduced into a square arena (55 × 55 × 40 cm) and allowed to explore freely for 15 min under dim white lighting (10 lx). Locomotor parameters, including total distance traveled and distance traveled within the central zone per minute (in meters), were automatically recorded and analyzed using Any-Maze tracking software (v4.72; Stoelting Co., Wood Dale, IL, USA). The frequency and duration of rearing and grooming were manually scored offline from video recordings using Solomon Coder software (v17.03.22) [34]. Distance traveled, rearing, and grooming were used as measures of spontaneous exploration and unconditioned anxiety. Behavioral analyses were performed by two independent experimenters blinded to group allocation, who assessed animals sequentially using code identifiers.

### 2.4. Serum Analyses

Trunk blood serum was obtained and analyzed as described in Vindas-Smith et al. (2025) [27]. Glucose, uric acid, total cholesterol (TC), high-density lipoprotein cholesterol (HDL), non-HDL cholesterol (TC—HDL), and triglycerides (TG) were quantified using an automated clinical chemistry analyzer (Roche Cobas^®^ c501; Roche Diagnostics, Indianapolis, IN, USA). All values are reported in mg/dL.

### 2.5. Brain Tissue Collection and Analyses

For neurochemical and expression analyses, brains were rapidly extracted, and the DS, NAc, HPC, and mPFC were dissected according to previously described procedures [34,35]. Tissue sampling followed an alternating right–left hemispheric approach to ensure balanced representation across analyses. All brain samples were stored at −80 °C until further processing.

#### 2.5.1. Neurotransmitter Contents

Monoamines and amino acids were quantified using high-performance liquid chromatography (HPLC) with electrochemical detection and reverse-phase HPLC with fluorescence detection, respectively (Agilent Technologies, Santa Clara, CA, USA), following previously described protocols [36]. Selected brain samples were analyzed for ex vivo levels of dopamine (DA), 3,4-dihydroxyphenylacetic acid (DOPAC), homovanillic acid (HVA), 5-hydroxytryptamine (5-HT), 5-hydroxyindoleacetic acid (5-HIAA), norepinephrine (NE), as well as glutamate (Glu), glutamine (Gln), and gamma-aminobutyric acid (GABA). Dopamine and 5-HT turnover were estimated as the ratios DOPAC/DA and 5-HIAA/5-HT, respectively. Neurochemical concentrations were measured as ng per mg of wet tissue weight and are expressed relative to the Control group.

#### 2.5.2. mRNA Isolation and Levels

Brain tissues were homogenized by sonication in 300 μL of TRIzol^®^ (Invitrogen™, Thermo Fisher Scientific, Waltham, MA, USA). RNA isolation and subsequent reverse transcription were performed according to a previously published protocol [36]. Primer sequences for mRNA quantification of *Bdnf*, *Ntrk2*, *Creb1*, *Dnmt3a*, and *Crf* were reported previously [27], whereas primers for *Hdac5* (Forward: 5′-TCGCTGAGAACGGCTTTACTGGC-3′; Reverse: 5′-GCAGAGAAGGAGACGTGTAGAGGC-3′) were designed using Primer3 v4.1.0 (https://primer3.ut.ee/) and OligoAnalyzer v3.1 (https://www.idtdna.com/pages/tools/oligoanalyzer). mRNA expression levels were determined using the comparative threshold cycle (Ct) method, with hypoxanthine phosphoribosyltransferase 1 (*Hprt1*) serving as the reference gene and analyzed using Rotor-Gene Q Series Software v2.0.2 (Qiagen, Hilden, Germany), as described previously [34]. Gene expression data were calculated as mean 2^−∆Ct^ values and expressed relative to the Control group.

#### 2.5.3. Protein Extraction and Western Blot

After RNA extraction with TRIzol^®^, proteins were isolated from the remaining organic phase according to the manufacturer’s instructions, with modifications described by Kopec et al. (2017) [37]. Briefly, after protein precipitation, samples were homogenized in lysis buffer (20 mM EDTA, 140 mM NaCl, 2% SDS, 100 mM Tris at pH 8.0) supplemented with the cOmplete™ protease inhibitor cocktail (Roche Diagnostics, Rotkreuz, Switzerland) and Pierce™ phosphatase inhibitor tablets (Thermo Fisher Scientific, Waltham, MA, USA). Samples were incubated at 50 °C for 2 h and then centrifuged for 5 min at 7600× *g* to collect the protein-containing supernatants. All procedures were performed on ice unless otherwise specified. Total protein concentrations were determined using a bicinchoninic acid (BCA) protein assay kit (Thermo Micro BCA Protein Assay, Thermo Fisher Scientific), following the manufacturer’s instructions, and measured with a NanoDrop™ 2000/2000c spectrophotometer (Thermo Fisher Scientific). 

Equal amounts of protein (30 μg) were mixed with Laemmli buffer 1× (62.5 mM Tris at pH 6.8, 2% SDS, 10% glycerol, 0.01% bromophenol blue, 5% β-mercaptoethanol), heated at 95 °C for 5 min, and separated by SDS-PAGE on 12% or 15% gels (the latter for CREB detection) at 120 V for 2 h. Proteins were then electrotransferred onto a nitrocellulose membrane (Bio-Rad, Hercules, CA, USA) at 80 V for 2 h. Protein transfer efficiency was evaluated by staining the membranes with Ponceau S solution at room temperature. Membranes were blocked with 5% non-fat milk in Tris-buffered saline containing 0.05% Tween 20 (1× TBST) for 1 h at room temperature and subsequently incubated overnight at 4 °C with rabbit monoclonal primary antibodies from Abcam (Cambridge, UK): anti-BDNF (1:500; ab108319), anti-CREB (1:2000; ab32515), and anti-phospho-CREB (Ser133) (pCREB; 1:1000; ab32096). Rabbit polyclonal anti-HPRT (1:1500; ab10479) was used as a loading control. All primary antibodies were diluted in TBST. After seven washes in TBST (5 min each), membranes were incubated for 1 h at room temperature with an HRP-conjugated goat anti-rabbit IgG (H&L) secondary antibody (Abcam, ab205718) diluted in TBST containing 3% non-fat milk (1:5000 for BDNF, 1:10,000 for CREB and pCREB, and 1:15,000 for HPRT). Immunoreactive bands were detected using Pierce™ ECL Western Blotting Substrate (Thermo Fisher Scientific) and visualized with a ChemiDoc Imaging System v6.1 (Bio-Rad). Densitometric analyses were performed using Image Lab software v6.1.0 (Bio-Rad). Protein levels were normalized to HPRT, and pCREB levels were normalized to total CREB. To account for inter-blot variability, a pooled homogenate sample was included in each gel and used as an inter-blot normalization control. Data were expressed relative to the Control group. HPC proteins were not detected in one sample from the CAFP group due to technical issues during processing; therefore, this sample was excluded from the analysis.

### 2.6. Statistical Analysis

Each animal was considered an experimental unit. Weekly mean daily food intake and body weight per animal, as well as outcomes from OFT and SPT, were analyzed using a one-way mixed multivariate analysis of covariance (MANCOVA), with Week or Test as the within-subject factor and Access Pattern (Control, CAFP, CAFU, and CAFC) as the between-subject factor. The dam was included as a covariate in all analyses. Overall mean weekly food intake and body weight, along with mean adiposity, serum, behavioral, neurochemical, and molecular outcomes, were analyzed using one-way ANCOVA with Access Pattern as the between-subject factor. When sphericity was violated (Mauchly’s test), Greenhouse–Geisser corrections were applied. Planned pairwise comparisons were performed using Reverse Helmert and Simple contrasts. Data are presented as estimated marginal means + SEM. Effect sizes were reported as partial eta squared (η^2^_p_) for main effects and Cohen’s *d* for pairwise comparisons. Statistical analyses were performed using IBM^®^ SPSS Statistics (v25.0; IBM Corp., Armonk, NY, USA). Statistical significance was set at *p* < 0.05. See Supplementary Information for details.

## 3. Results

### 3.1. Effects on Food Intake Parameters

**Figure 2:**
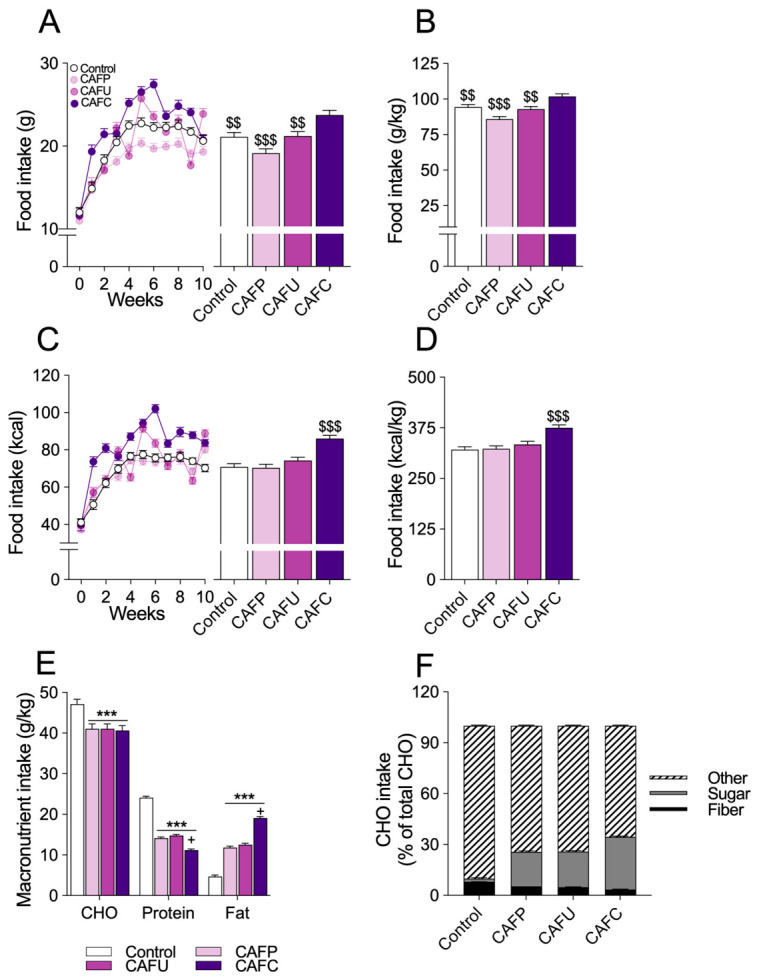
Effects of cafeteria diet access patterns on food intake behavior over the 10-week feeding period. (**A**) Weekly mean daily food intake per animal (g; left panel) and overall mean food intake (right panel). (**B**) Overall mean food intake normalized to body weight (g/kg). (**C**) Weekly mean daily energy intake per animal (kcal; left panel) and overall mean energy intake (right panel). (**D**) Overall mean energy intake normalized to body weight (kcal/kg). (**E**) Overall mean macronutrient intake normalized to body weight (g/kg). (**F**) Sugar and fiber intake relative to total carbohydrates intake. Overall mean values represent averages across the 10-week. Data are presented as estimated marginal means + SEM from ANCOVAs (*n* = 10 animals/group). Symbols denote planned contrasts: CAFC vs. other groups: $$ *p* < 0.01, $$$ *p* < 0.001; Control vs. all CAF-access groups: *** *p* < 0.001; CAFC vs. intermittent-access groups: + *p* < 0.05. See main text for details. Exclusively chow-fed rats constitute the Control group. CAFP: cafeteria predictable intermittent-access group; CAFU: cafeteria unpredictable intermittent-access group; CAFC: cafeteria continuous-access group; CHO: total carbohydrates.

Food intake (g) and energy intake (kcal) varied significantly across the 10-week protocol (Week g: *F*_9, 315_ = 22.31, *p* = 0.0001, η^2^_p_ = 0.39; Week kcal: *F*_9, 315_ = 18.47, *p* = 0.0001, η^2^_p_ = 0.35; Figure 2A,C, left panels). All rats increased their intake throughout the weeks, with the chow-fed and CAFP rats showing a steady pattern from week 4 onward. The CAFC group exhibited an escalating intake, whereas the CAFU group showed an irregular pattern starting at week 3, characterized by peaks followed by abrupt reductions, with peak values comparable to those observed in the CAFC group (Figure 2A,C, left panels). Consequently, significant Week × Access Pattern interactions were observed (g: *F*_27, 315_ = 13.84, *p* = 0.0001, η^2^_p_ = 0.54; kcal: *F*_27, 315_ = 14.81, *p* = 0.0001, η^2^_p_ = 0.56). A significant main effect of Access Pattern was observed, with the CAFC group consuming more food (g: *F*_3, 35_ = 12.50, *p* = 0.0001, η^2^_p_ = 0.52) and energy (kcal: *F*_3, 35_ = 17.20, *p* = 0.0001, η^2^_p_ = 0.60) than the other groups (Figure 2A,C, right panels). These effects persisted when food intake was normalized to body weight (g: *F*_3, 35_ = 13.13, *p* = 0.0001, η^2^_p_ = 0.53; Figure 2B; kcal: *F*_3, 35_ = 11.88, *p* = 0.0001, η^2^_p_ = 0.51; Figure 2D).

All groups with access to the CAF diet consumed less carbohydrates (CHO) and protein and more fat than the Control group (all *p*-values < 0.0001; Figure 2E). In contrast, groups with intermittent access to the CAF diet consumed more protein (*t*_35_ = 9.35, *p* = 0.0001, *d* = 3.16) and less fat (*t*_35_ = −15.87, *p* = 0.0001, *d* = −5.36) than the CAFC animals (Figure 2E). While CHO intake did not differ among CAF groups, the proportions of sugar and fiber relative to total CHO intake differed (Figure 2F). CAFC animals showed lower fiber and higher sugar intake (*t*_35_ = −20.26, *p* = 0.0001, *d* = −6.85; *t*_35_ = 17.52, *p* = 0.0001, *d* = 5.92, respectively) than rats with intermittent access to the CAF diet. CAFP and CAFU rats differed in fiber intake (*t*_35_ = 4.94, *p* = 0.0001, *d* = 1.67), with CAFU showing lower intake. Despite consuming more total CHO, the Control group exhibited lower sugar and higher fiber intake than the other groups (*t*_35_ = −40.12, *p* = 0.0001, *d* = −13.56; *t*_35_ = 46.88, *p* = 0.0001, *d* = 15.84, respectively).

**Figure 3:**
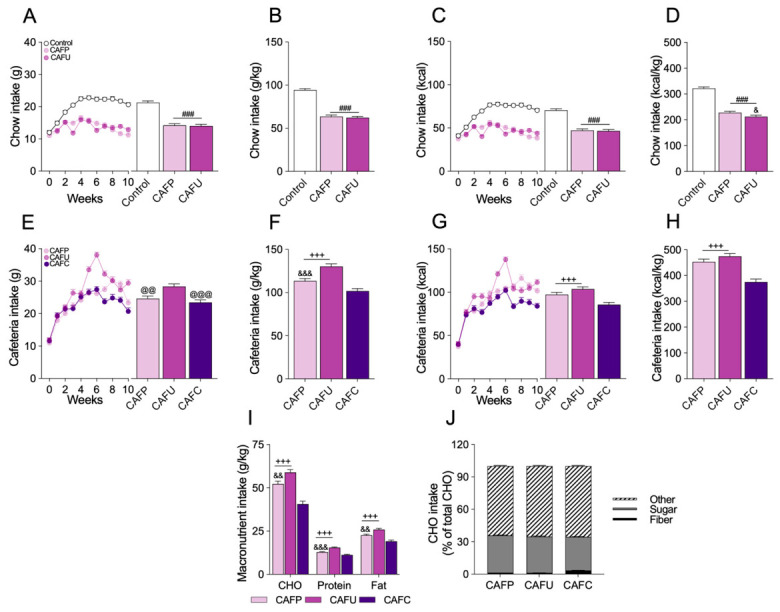
Effects of diet access pattern on food intake behavior during chow-only and CAF days over the 10-week feeding period. (**A**) Weekly mean daily chow intake per animal (g; left panel) and overall mean chow intake (right panel). (**B**) Overall mean chow intake normalized to body weight (g/kg). (**C**) Weekly mean daily chow-derived energy intake per animal (kcal; left panel) and overall mean chow-derived energy intake (right panel). (**D**) Overall mean chow-derived energy intake normalized to body weight (kcal/kg). (**E**) Weekly mean daily CAF diet intake per animal (g; left panel) and overall mean CAF diet intake (right panel). (**F**) Overall mean CAF diet intake normalized to body weight (g/kg). (**G**) Weekly mean daily CAF diet-derived energy intake per animal (kcal; left panel) and overall mean CAF diet-derived energy intake (right panel). (**H**) Overall mean CAF diet-derived energy intake normalized to body weight (kcal/kg). (**I**) Overall mean macronutrient intake normalized to body weight during CAF days. (**J**) Sugar and fiber intake relative to total carbohydrate intake during CAF days. Overall mean values represent averages across the weeks within each feeding condition. Data are presented as estimated marginal means + SEM derived from ANCOVAs (*n* = 10 animals/group). Symbols denote planned contrasts: Control vs. intermittent-access groups: ### *p* < 0.001; CAFU vs. CAFP and CAFC groups: @@ *p* < 0.01, @@@ *p* < 0.001; CAFC vs. intermittent-access groups: +++ *p* < 0.001; CAFP vs. CAFU: & *p* < 0.05, && *p* < 0.01, &&& *p* < 0.001. See main text for details. Exclusively chow-fed rats correspond to the Control group. CAF: cafeteria diet; CAFP: cafeteria predictable intermittent-access group; CAFU: cafeteria unpredictable intermittent-access group; CAFC: cafeteria continuous-access group; CHO: total carbohydrates.

The analysis of food intake behavior during chow-only and CAF days revealed distinct intake patterns as a function of intermittency and predictability (Figure 3). On chow-only days, the CAFU group displayed a fluctuating pattern of intake similar to that shown in Figure 2A (left panel), with peaks and subsequent declines from week 2 onward, and peak intake matched that of the CAFP group (Figure 3A, left panel). The CAFP group showed a steady pattern with a tendency to decrease from week 3 onward. Across weeks, the intermittent-access groups had significantly lower chow intake than the Control group (Week × Access Pattern: *F*_9.75, 126.80_ = 18.68, *p* = 0.0001, η^2^_p_ = 0.59), even as early as week 1 (all *p*-values < 0.01; Figure 3A, left panel).

A main effect of Access Pattern was observed for overall mean chow intake and chow intake relative to body weight (*F*_2, 26_ = 64.63, *p* = 0.0001, η^2^_p_ = 0.83; *F*_2, 26_ = 136.73, *p* = 0.0001, η^2^_p_ = 0.91, respectively), with intermittent-access groups consuming approximately 33% less chow than the Control group (g: *t*_26_ = −11.37, *p* = 0.0001, *d* = −4.46; Figure 3A, right panel; g/kg: *t*_26_ = −16.52, *p* = 0.0001, *d* = −6.48; Figure 3B). Similar results were observed for chow-derived energy intake (Figure 3C,D). Intermittent-access groups differed only when chow-derived energy intake was normalized to body weight, with the CAFU group exhibiting lower intake (*t*_26_ = −2.14, *p* = 0.0001, *d* = −0.84; Figure 3D). This effect appears to be associated with differences in body weight during adolescence, as suggested by significant differences in normalized chow-derived energy intake during weeks 1–5 (*p* = 0.001; Figure S1H, left panel), but not during weeks 6–10 (*p* = 0.663; Figure S1H, right panel).

During CAF days, intermittent-access groups consumed twice as much food as on chow-only days (g: CAFP: 24.7 ± 0.8 vs. 13.9 ± 0.5, CAFU: 28.4 ± 0.8 vs. 13.7 ± 0.5; kcal: CAFP: 97.7 ± 2.5 vs. 47.5 ± 1.7, CAFU: 103.9 ± 2.5 vs. 46.9 ± 1.7). Intake increased across weeks in all CAF-fed rats (Week: *F*_9, 234_ = 20.65, *p* = 0.0001, η^2^_p_ = 0.44; Figure 3E, left panel), following an inverted U-shaped pattern. The CAFU group showed the most pronounced escalation in intake, followed by the CAFP and then the CAFC group (Week × Access Pattern: *F*_18, 234_ = 16.11, *p* = 0.0001, η^2^_p_ = 0.55). A main effect of Access Pattern was observed (*F*_2, 26_ = 10.55, *p* = 0.0004, η^2^_p_ = 0.45), indicating a higher CAF diet intake in CAFU rats compared with both CAFP (*t*_26_ = 3.32, *p* = 0.003, *d* = 1.30) and CAFC animals (*t*_26_ = 4.41, *p* = 0.0002, *d* = 1.73), with no differences between the two latter groups (Figure 3E, right panel). As shown in Figure 3E (left panel), the CAFP group increased its intake relative to the CAFC group from week 6 onward. Accordingly, both intermittent-access groups differed from the CAFC group during weeks 6–10 (*p* = 0.0001; Figure S1I, right panel).

When normalized to body weight, both intermittent-access groups consumed more CAF food than the continuous-access group (*t*_26_ = 5.84, *p* = 0.0001, *d* = 2.29), with the CAFU group exhibiting higher intake than the CAFP group (*t*_26_ = 4.22, *p* = 0.0003, *d* = 1.66) (Figure 3F). Relative to the CAFC group, the CAFP and CAFU groups consumed approximately 12% and 28% more CAF food, respectively. When CAF diet-derived energy intake was analyzed, no differences were observed between intermittent-access groups (Figure 3G,H), whereas both groups consumed more energy than the CAFC group (kcal: *t*_26_ = 4.75, *p* = 0.0001, *d* = 1.86; kcal/kg: *t*_26_ = 6.62, *p* = 0.0001, *d* = 2.60). The CAFU group consumed more energy than the CAFP group during CAF days across weeks 1–5 (Figure S1K, left panel), although this effect disappeared during weeks 6–10, when CAFP rats increased their CAF diet intake relative to the CAFC group (Figure 3G, left panel; Figure S1K, right panel).

Finally, on CAF days, macronutrient intake differed among groups (CHO: *F*_2, 26_ = 32.25, *p* = 0.0001, η^2^_p_ = 0.71; protein: *F*_2, 26_ = 41.81, *p* = 0.0001, η^2^_p_ = 0.76; fat: *F*_2, 26_ = 24.04, *p* = 0.0001, η^2^_p_ = 0.65) (Figure 3I). CHO (*t*_26_ = 7.49, *p* = 0.0001, *d* = 2.94), protein (*t*_26_ = 7.18, *p* = 0.0001, *d* = 2.82), and fat (*t*_26_ = 6.04, *p* = 0.0001, *d* = 2.37) intake were higher in intermittent-access groups than in the CAFC group. Moreover, unpredictable access to the CAF diet resulted in higher intake values than the CAFP group (CHO: *t*_26_ = 2.91, *p* = 0.007, *d* = 1.14; protein: *t*_26_ = 5.67, *p* = 0.0001, *d* = 2.22; fat: *t*_26_ = 3.41, *p* = 0.002, *d* = 1.34). Intermittent-access groups consumed less fiber (*t*_26_ = −32.75, *p* = 0.0001, *d* = −12.85) and more sugar (*t*_26_ = 4.23, *p* = 0.0003, *d* = 1.66) than the CAFC group (Figure 3J). CAFU rats consumed less fiber than CAFP rats (*t*_26_ = −2.82, *p* = 0.009, *d* = −1.11), whereas sugar intake did not differ significantly between intermittent-access groups.

### 3.2. Body Weight Gain and Fat Accumulation

**Figure 4:**
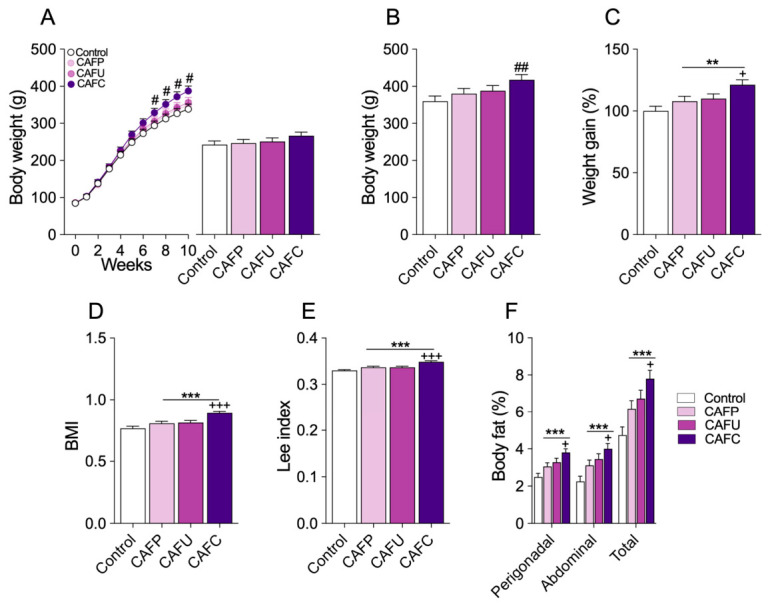
Effects of cafeteria diet access patterns on rodent biometric indices and body fat mass. (**A**) Weekly mean daily body weight per animal (g; left panel) and overall mean body weight (right panel). (**B**) Final body weight. (**C**) Weight gain from week 1 to 10 expressed as a percentage relative to the Control group. (**D**) Body mass index calculated as g of body weight/animal length in cm^2^. (**E**) Lee index calculated as ^3^√g of body weight/animal length in cm. (**F**) Perigonadal, abdominal, and total white adipose tissue depots expressed as percentages of total body weight. Data are presented as estimated marginal means + SEM from ANCOVAs (*n* = 10 animals/group). Symbols denote planned contrasts: Control vs. CAFC group: # *p* < 0.05, ## *p* < 0.01; Control vs. all CAF-access groups: ** *p* < 0.01, *** *p* < 0.001; CAFC vs. intermittent-access groups: + *p* < 0.05, +++ *p* < 0.001. See main text for details. Exclusively chow-fed rats constitute the Control group. CAFP: cafeteria predictable intermittent-access group; CAFU: cafeteria unpredictable intermittent-access group; CAFC: cafeteria continuous-access group; BMI: body mass index.

As expected for rats at this developmental stage, body weight increased over time across all groups (Week: *F*_1.21, 42.44_ = 537.15, *p* = 0.0001, η^2^_p_ = 0.94; Figure 4A, left panel). The CAFC group exhibited a higher growth rate (Week × Access Pattern: *F*_3.64, 42.44_ = 4.53, *p* = 0.005, η^2^_p_ = 0.28). Week-by-week between-group comparisons revealed significant differences between the CAFC and Control groups from week 7 onward (all *p*-values < 0.05); however, no significant between-group differences were observed (*p* = 0.38; Figure 4A, right panel). Final body weight and weight gain differed among groups (*F*_3, 35_ = 2.88, *p* = 0.049, η^2^_p_ = 0.20; *F*_3, 35_ = 4.83, *p* = 0.006, η^2^_p_ = 0.29, respectively). Only the CAFC group differed from the Control group in final body weight (*t*_35_ = 2.90, *p* = 0.006, *d* = 0.98; Figure 4B), while weight gain differed between the Control and all CAF-access groups (*t*_35_ = −2.81, *p* = 0.008, *d* = −0.95), with the CAFC group also differing from the intermittent-access groups (*t*_35_ = 2.54, *p* = 0.016, *d* = 0.86) (Figure 4C).

Rodent anthropometric indices of obesity and body composition revealed more robust differences (all *p*-values < 0.001). Access to the CAF diet, independent of access schedule, resulted in higher BMI (*t*_35_ = 3.55, *p* = 0.001, *d* = 1.20; Figure 4D) and Lee index (*t*_35_ = 3.67, *p* = 0.0002, *d* = 1.24; Figure 4E) compared with Control rats. Moreover, the CAFC group exhibited higher values than the intermittent-access groups (BMI: *t*_35_ = 3.86, *p* = 0.0005, *d* = 1.30; Lee index: *t*_35_ = 4.00, *p* = 0.0001, *d* = 1.35). These differences were also reflected in body fat accumulation (Figure 4F). All CAF-access groups showed greater perigonadal (*t*_35_ = 3.72, *p* = 0.0007, *d* = 1.26), abdominal (*t*_35_ = 3.90, *p* = 0.0004, *d* = 1.32), and total WAT (*t*_35_ = 4.25, *p* = 0.0001, *d* = 1.44) than chow-fed Control rats. The CAFC group had greater body fat accumulation than the intermittent-access groups (perigonadal: *t*_35_ = 2.52, *p* = 0.017, *d* = 0.85; abdominal: *t*_35_ = 2.07, *p* = 0.046, *d* = 0.70; total WAT: *t*_35_ = 2.51, *p* = 0.017, *d* = 0.85). In contrast, liver weight was similar across groups (*p* = 0.085).

### 3.3. Serum Metabolic Parameters

**Figure 5:**
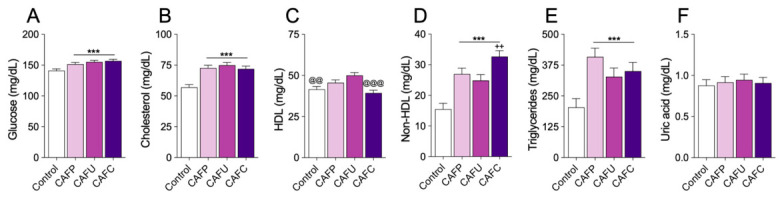
Effects of cafeteria diet access patterns on serum metabolic parameters. (**A**) Glucose levels. (**B**) Total cholesterol levels. (**C**) High-density lipoprotein cholesterol levels. (**D**) Non-HDL cholesterol levels calculated as (Total cholesterol—HDL). (**E**) Triglyceride levels. (**F**) Uric acid levels. Data are expressed in mg/dL and presented as estimated marginal means + SEM from ANCOVAs (*n* = 10 animals/group). Symbols denote planned contrasts: Control vs. all CAF-access groups: *** *p* < 0.001; CAFU vs. Control and CAFC groups: @@ *p* < 0.01, @@@ *p* < 0.001; CAFC vs. intermittent-access groups: ++ *p* < 0.01. See main text for details. Exclusively chow-fed rats constitute the Control group. CAFP: cafeteria predictable intermittent-access group; CAFU: cafeteria unpredictable intermittent-access group; CAFC: cafeteria continuous-access group; HDL: high-density lipoprotein cholesterol.

Significant differences were observed across all measured serum parameters (glucose: *F*_3, 35_ = 7.32, *p* = 0.0006, η^2^_p_ = 0.39; TC: *F*_3, 35_ = 13.17, *p* = 0.0001, η^2^_p_ = 0.53; HDL: *F*_3, 35_ = 7.86, *p* = 0.0004, η^2^_p_ = 0.40; non-HDL: *F*_3, 35_ = 14.12, *p* = 0.0001, η^2^_p_ = 0.55; TG: *F*_3, 35_ = 5.94, *p* = 0.002, η^2^_p_ = 0.34) (Figure 5A–E), except for uric acid levels (Figure 5F). All CAF-access groups showed significantly higher levels of glucose (*t*_35_ = 4.46, *p* = 0.0001, *d* = 1.51), TC (*t*_35_ = 6.21, *p* = 0.0001, *d* = 2.10), non-HDL (*t*_35_ = 5.78, *p* = 0.0001, *d* = 1.95), and TG (*t*_35_ = 3.88, *p* = 0.0004, *d* = 1.31) than the Control group. The CAFP and CAFU groups showed lower non-HDL levels than the CAFC group (*t*_35_ = −2.90, *p* = 0.006, *d* = −0.98). Regarding HDL levels, the CAFU group had the highest values, differing from both the CAFC (*t*_35_ = 4.50, *p* = 0.0001, *d* = 1.52) and Control (*t*_35_ = 3.56, *p* = 0.001, *d* = 1.20) groups.

### 3.4. Reward-Related Behavior and Locomotor Activity

**Figure 6:**
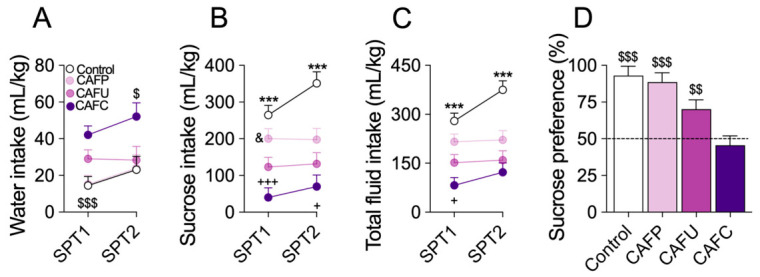
Effects of cafeteria diet access patterns on sucrose preference. (**A**) Water intake in mL normalized to body weight. (**B**) Sucrose intake normalized to body weight. (**C**) Total fluid intake normalized to body weight. (**D**) Cumulative sucrose preference expressed as a percentage of total fluid intake. Data are presented as estimated marginal means + SEM from ANCOVAs (*n* = 10 animals/group). Symbols denote planned contrasts: CAFC vs. other groups: $ *p* < 0.05, $$ *p* < 0.01, $$$ *p* < 0.001 (except vs. CAFU for water intake in SPT1); Control vs. all CAF-access groups: *** *p* < 0.001; CAFC vs. intermittent-access groups: + *p* < 0.05, +++ *p* < 0.001; CAFP vs. CAFU: & *p* < 0.05. See main text for details. Exclusively chow-fed rats constitute the Control group. CAFP: cafeteria predictable intermittent-access group; CAFU: cafeteria unpredictable intermittent-access group; CAFC: cafeteria continuous-access group; SPT1 and SPT2: sucrose preference tests 1 and 2.

No significant differences were observed between the two test sessions in either the SPT or OFT (all *p*-values > 0.05). In the SPT, a significant main effect of Access Pattern was detected for water intake (*F*_3, 35_ = 5.73, *p* = 0.003, η^2^_p_ = 0.33; Figure 6A), sucrose intake (*F*_3, 35_ = 15.69, *p* = 0.0001, η^2^_p_ = 0.57; Figure 6B), total fluid intake (*F*_3, 35_ = 14.98, *p* = 0.0001, η^2^_p_ = 0.56; Figure 6C), and sucrose preference (*F*_3, 35_ = 11.38, *p* = 0.0001, η^2^_p_ = 0.49; Figure 6D). All groups showed a preference for sucrose over water (range: 70.2–93.1%), except for the CAFC group, which had a sucrose preference significantly below 50% and lower than that of the other groups (*t*_35_ = −2.72–−5.25, *p* = 0.0001–0.01, *d* = −0.92–−1.77). Sucrose preference showed a statistically significant inverse linear relationship (*r* = −0.674, *p* = 0.0001; Figure 6D), with no significant deviation from linearity (*p* = 0.313).

**Figure 7:**
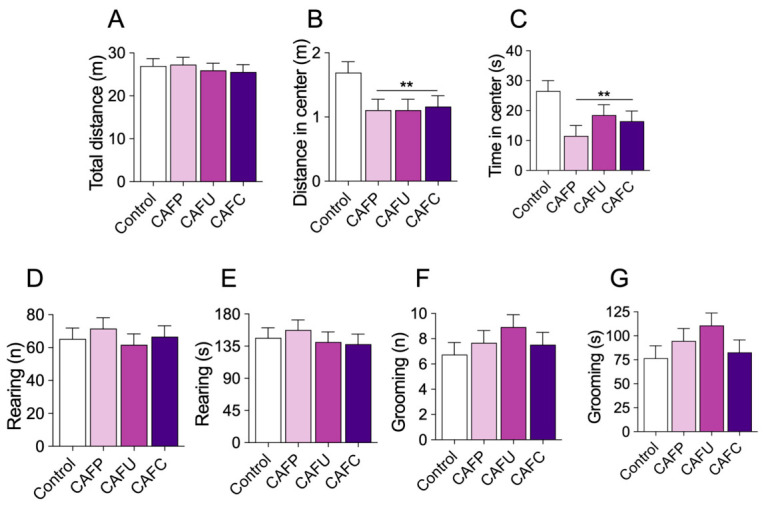
Effects of cafeteria diet access patterns on open field test behaviors. (**A**) Total distance traveled in meters. Distance traveled (**B**) and time spent (**C**) in the center zone. (**D**) Rearing frequency. (**E**) Rearing duration. (**F**) Grooming frequency (**G**) Grooming duration. Data are presented as estimated marginal means + SEM from ANCOVAs (*n* = 10 animals/group). Symbols denote planned contrasts: Control vs. all CAF-access groups: ** *p* < 0.01. See main text for details. Exclusively chow-fed rats constitute the Control group. CAFP: cafeteria predictable intermittent-access group; CAFU: cafeteria unpredictable intermittent-access group; CAFC: cafeteria continuous-access group.

For locomotor activity and unconditioned anxiety-like behavior, no significant between-group differences were observed in total distance traveled (Figure 7A), rearing (Figure 7D,E), or grooming behaviors (Figure 7F,G) in the OFT. However, a significant main effect of Diet was detected for both total distance traveled and time spent in the center zone (Figure 7B,C). Specifically, CAF diet rats traveled less distance and spent less time in the center than chow-fed Control rats (distance traveled: *F*_1, 37_ = 8.74, *p* = 0.005, η^2^_p_ = 0.19; center time: *F*_1, 37_ = 7.71, *p* = 0.009, η^2^_p_ = 0.17). This effect remained statistically significant after controlling for body fat mass (distance traveled: *F*_1, 36_ = 4.59, *p* = 0.039, η^2^_p_ = 0.11; center time: *F*_1, 36_ = 5.51, *p* = 0.025, η^2^_p_ = 0.13).

### 3.5. Effects of CAF Diet Exposure on Neurotransmitter Contents

**Figure 8:**
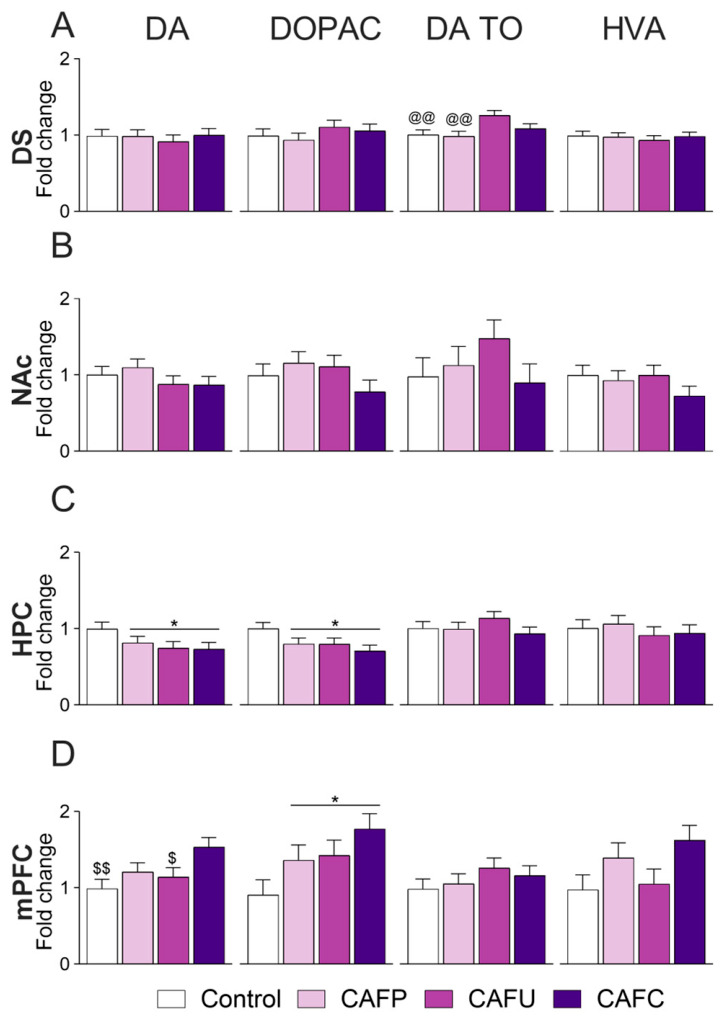
Effects of diet access patterns on dopamine metabolism in reward-related brain regions. Dopamine, DOPAC, dopamine turnover, and HVA levels in the (**A**) dorsal striatum, (**B**) nucleus accumbens, (**C**) hippocampus, and (**D**) medial prefrontal cortex. Data are expressed relative to the Control group and presented as estimated marginal means + SEM from ANCOVAs (*n* = 10 animals/group). Symbols denote planned contrasts: CAFU vs. CAFP and Control groups: @@ *p* < 0.01; Control vs. all CAF-access groups: * *p* < 0.05; CAFC vs. CAFU and Control groups: $ *p* < 0.05, $$ *p* < 0.01. See main text for details. Exclusively chow-fed rats constitute the Control group. CAFP: cafeteria predictable intermittent-access group; CAFU: cafeteria unpredictable intermittent-access group; CAFC: cafeteria continuous-access group; DA: dopamine; DOPAC: 3,4-dihydroxyphenylacetic acid; DA TO: dopamine turnover; HVA: homovanillic acid.

Hippocampal Glu levels were significantly affected by Access Pattern (*F*_3, 35_ = 3.51, *p* = 0.025, η^2^_p_ = 0.23; Table S3). The CAFC group showed lower levels than the Control group (*t*_35_ = −3.22, *p* = 0.003, *d* = −1.01), with no differences compared to the intermittent-access groups. Regarding dopaminergic markers, a significant main effect of Access Pattern was observed on DA turnover in the DS (*F*_3, 35_ = 3.57, *p* = 0.024, η^2^_p_ = 0.23; Figure 8A) and on DA levels in the mPFC (*F*_3, 35_ = 3.57, *p* = 0.024, η^2^_p_ = 0.23; Figure 8D). The CAFU group exhibited higher DA turnover in the DS than the Control (*t*_35_ = 2.73, *p* = 0.01, *d* = 0.92) and CAFP groups (*t*_35_ = 2.92, *p* = 0.006, *d* = 0.99), with no significant differences compared to the CAFC group (*p* = 0.07). In the mPFC, DA levels were higher in the CAFC group than in the Control (*t*_35_ = 3.13, *p* = 0.003, *d* = 1.06) and CAFU (*t*_35_ = 2.25, *p* = 0.029, *d* = 0.76) groups. Additionally, a main effect of Diet was observed on DA (*F*_1, 37_ = 5.75, *p* = 0.022, η^2^_p_ = 0.14; Figure 8C) and DOPAC levels (*F*_1, 37_ = 6.81, *p* = 0.013, η^2^_p_ = 0.16) in the HPC, and on DOPAC levels (*F*_1, 37_ = 7.38, *p* = 0.01, η^2^_p_ = 0.19) in the mPFC. All CAF-access groups showed lower DA and DOPAC levels in the HPC and higher DOPAC levels in the mPFC compared with the Control group. No significant between-group differences were observed in the NAc (Figure 8B) or in the other neurotransmitters (Table S3).

### 3.6. Effects of CAF Diet Exposure on mRNA and Protein Levels

**Figure 9:**
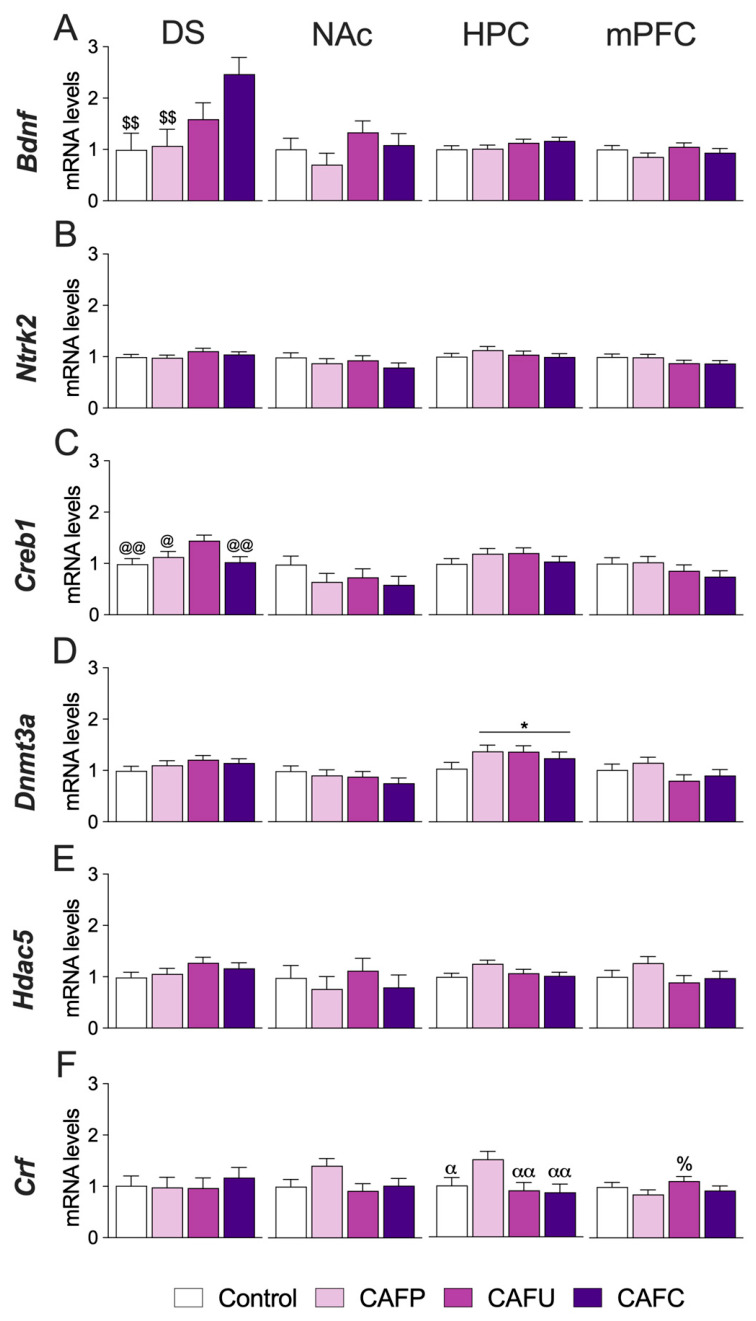
Effects of diet access patterns on mRNA levels in reward-related brain regions. (**A**) *Bdnf*, (**B**) *Ntrk2*, (**C**) *Creb1*, (**D**) *Dnmt3a*, (**E**) *Hdac5*, and (**F**) *Crf* in the dorsal striatum (DS), nucleus accumbens (NAc), hippocampus (HPC), and medial prefrontal cortex (mPFC). Data are expressed relative to the Control group and presented as estimated marginal means + SEM from ANCOVAs (*n* = 10 animals/group). Symbols denote planned contrasts: CAFC vs. CAFP and Control groups: $$ *p* < 0.01; CAFU vs. other groups: @ *p* < 0.05, @@ *p* < 0.01; Control vs. all CAF-access groups: * *p* < 0.05; CAFP vs. other groups: α *p* < 0.05, αα *p* < 0.01; CAFU vs. CAFC and CAFP groups: % *p* < 0.05. See main text for details. Exclusively chow-fed rats constitute the Control group. CAFP: cafeteria predictable intermittent-access group; CAFU: cafeteria unpredictable intermittent-access group; CAFC: cafeteria continuous-access group; *Bdnf*: brain-derived neurotrophic factor; *Ntrk2*: neurotrophic receptor tyrosine kinase 2; *Creb1*: cAMP response element-binding protein; *Crf*: corticotropin-releasing factor; *Dnmt3a*: DNA methyltransferase 3 alpha; *Hdac5*: histone deacetylase 5.

Regarding mRNA levels, a main effect of Access Pattern was observed for *Bdnf* (*F*_3, 35_ = 4.45, *p* = 0.009, η^2^_p_ = 0.28; Figure 9A) and *Creb1* (*F*_3, 35_ = 3.71, *p* = 0.02, η^2^_p_ = 0.24; Figure 9C) in the DS, as well as for *Crf* in the HPC (*F*_3, 35_ = 3.66, *p* = 0.021, η^2^_p_ = 0.24; Figure 9F). The CAFC group showed higher *Bdnf* mRNA levels in the DS than the Control (*t*_35_ = 3.25, *p* = 0.003, *d* = 1.10) and CAFP (*t*_35_ = 3.07, *p* = 0.004, *d* = 1.04) groups. For *Creb1* gene expression, only the CAFU group exhibited upregulated levels compared with the other groups (CAFU vs. Control: *t*_35_ = 3.00, *p* = 0.005, *d* = 1.01; CAFU vs. CAFP: *t*_35_ = 2.10, *p* = 0.045, *d* = 0.71; CAFU vs. CAFC: *t*_35_ = 2.76, *p* = 0.009, *d* = 0.93). Conversely, CAFP rats showed higher hippocampal *Crf* mRNA levels than the other groups (CAFP vs. Control: *t*_35_ = 2.32, *p* = 0.026, *d* = 0.78; CAFP vs. CAFU: *t*_35_ = 2.75, *p* = 0.009, *d* = 0.93; CAFP vs. CAFC: *t*_35_ = 2.91, *p* = 0.006, *d* = 0.98). An exclusive main effect of Diet was found for *Dnmt3a* expression in the HPC (Figure 9D), with all CAF-access groups showing increased mRNA levels compared with controls (*F*_1, 37_ = 4.61, *p* = 0.038, η^2^_p_ = 0.11). Additionally, a main effect of Predictability was observed for *Crf* mRNA levels in the mPFC, with both groups having predictable access to the CAF diet exhibiting lower levels than the CAFU group (*F*_1, 27_ = 4.40, *p* = 0.046, η^2^_p_ = 0.14). No significant between-group differences were observed in *Ntrk2* (Figure 9B) or *Hdac5* (Figure 9E) mRNA levels. In addition, no effects were observed in the NAc.

**Figure 10:**
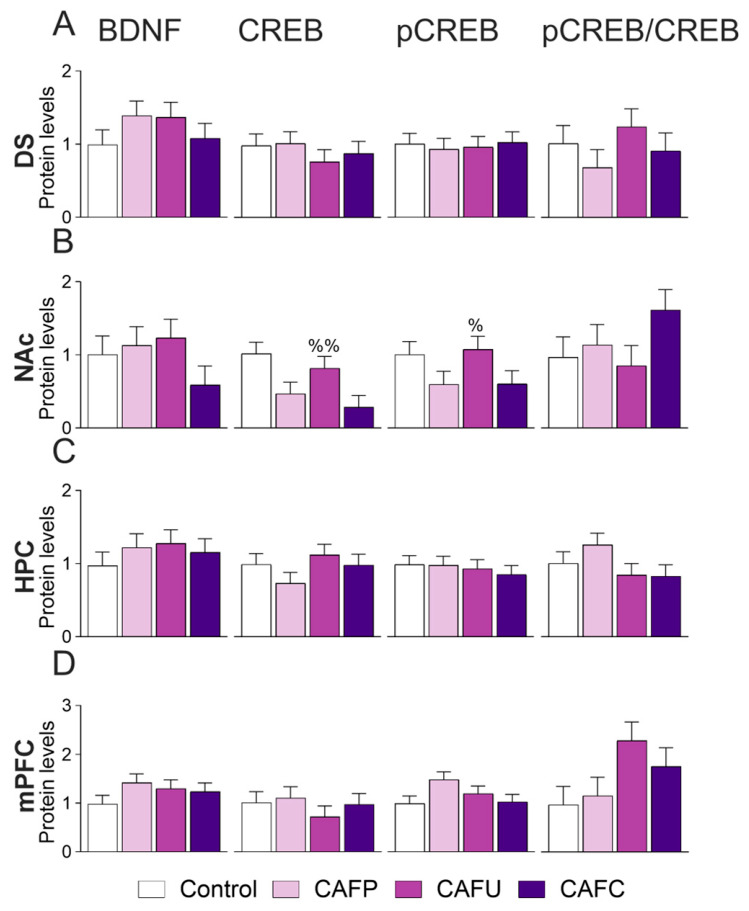
Effects of diet access patterns on neuroplasticity-related protein levels in reward brain regions. BDNF, CREB, pCREB, and pCREB/CREB levels in the (**A**) dorsal striatum (DS), (**B**) nucleus accumbens (NAc), (**C**) hippocampus (HPC), and (**D**) medial prefrontal cortex (mPFC). Samples were run on separate blots processed in parallel and normalized using an inter-blot control. Data are expressed relative to the Control group and presented as estimated marginal means + SEM from ANCOVAs (*n* = 9–10 animals/group). Symbols denote planned contrasts: CAFU vs. CAFC and CAFP groups: % *p* < 0.05, %% *p* < 0.01. See main text for details. Exclusively chow-fed rats constitute the Control group. CAFP: cafeteria predictable intermittent-access group; CAFU: cafeteria unpredictable intermittent-access group; CAFC: cafeteria continuous-access group. BDNF: brain-derived neurotrophic factor; CREB: cAMP response element-binding protein, pCREB: phospho-CREB.

Regarding protein levels, we detected a main effect of Predictability on CREB and pCREB levels in the NAc (*F*_1, 27_ = 10.46, *p* = 0.003, η^2^_p_ = 0.28 and *F*_1, 27_ = 6.21, *p* = 0.019, η^2^_p_ = 0.19, respectively; Figure 10B). The CAFC and CAFP groups exhibited lower levels compared with the CAFU group. No other significant between-group differences were observed.

## 4. Discussion

The present study investigated how the pattern and predictability of access to a palatable CAF diet influence feeding behavior, metabolic and neurobiological outcomes, and anxiety- and reward-related responses. Our results revealed that both exposure duration and access predictability play distinct roles in shaping these responses.

Our findings indicate that continuous exposure to the CAF diet induced sustained hyperphagia, in accordance with previous reports [27,31,38,39,40]. This is reflected in the highest overall food and energy intake across weeks compared with other groups, even after normalizing for body weight (Figure 2). This pattern is supported by animal studies showing that exposure to palatable or energy-dense diets induces neuroadaptations in the brain reward system [41,42,43,44]. These results may be explained theoretically by a sequence of alterations in the reward system. The constant availability of HPF—rich in sugar and fat—likely boosts dopamine in mesolimbic reward regions during the first weeks of exposure [26,45,46,47]. Over time, compensatory mechanisms may reduce reward sensitivity while sustaining high intake [41,43], thereby maintaining motivation despite lower or unchanged hedonic impact [48,49].

In addition to reward-related adaptations, overfeeding in CAFC rats may also involve alterations in homeostatic satiety mechanisms. The consumption of foods high in sugar and fat and low in protein and fiber is known to affect satiety signaling by reducing fullness and altering gut-derived hormones and other peripheral signals that normally suppress appetite [50,51,52]. This interpretation is also consistent with the protein-leverage hypothesis, which proposes that reduced dietary protein content promotes compensatory increases in overall food intake [53,54]. Consistent with this hypothesis, all CAF groups consumed less protein than the Control group, which may have contributed to increased intake of fat- and CHO-rich foods. Accordingly, CAFC animals showed the most pronounced increases in body weight, BMI, and Lee index, along with marked accumulation of body fat, especially in the abdominal depot (Figure 4). Overall, these findings suggest that alterations in reward-related mechanisms, along with reduced satiety, contributed to the increased food intake and adiposity observed in CAFC rats.

Our principal findings emerged from the analysis of food intake behavior on chow-only and CAF days (Figure 3). On chow-only days, animals with intermittent access consumed approximately one-third less chow than Control rats, with no differences between intermittent-access groups. In contrast, on CAF days, both intermittent groups overate relative to animals with continuous access, with the CAFU group showing the most pronounced escalation in intake after normalization to body weight (g/kg, Figure 3F). These results indicate that withdrawal from the palatable food induced hypophagia, whereas renewed access triggered a binge-like intake pattern, which was further amplified by the unpredictability of access to the preferred diet. This interpretation is consistent with previous research using limited-access models, in which intermittent access to HPFs (e.g., CAF diets, sugary chocolate-flavored pellets, high-fat or high-fat/high-sugar diets) produces episodic increases in intake on access days at the expense of reduced chow consumption during withdrawal [8,9,11,19,20,21,40,41,55,56]. Although unpredictable-access models have been less explored, similar outcomes have been reported [10,22,23].

It has been suggested that the reduction in intake on chow-only days may reflect a decreased motivational value of chow relative to the CAF diet, rather than solely adaptive homeostatic mechanisms dependent on prior hyperphagia for palatable food [9,57]. Our data support this view, as rats with intermittent access showed increased intake on CAF days that tended to exceed the reductions observed on chow-only days. This negative contrast in motivational salience between diets could also contribute to a transient state of negative affect, which may be relieved upon re-exposure to the CAF diet [55,56]. Overall, this view highlights the relevance of palatability and feeding schedule in promoting excessive food intake, particularly when access to the preferred diet is intermittent and unpredictable. The putative heightened motivational and reinforcing value associated with this pattern of access may involve the recruitment of the mesolimbic dopaminergic system [58].

A large body of evidence from human and animal studies indicates that intermittency can shift dopaminergic neuron activity from tonic to phasic firing, encoding positive reward prediction errors (i.e., the reward is better than expected), which are typically larger under conditions of uncertainty [59,60,61,62]. Under intermittent but predictable access, rats may learn the temporal dynamics of the feeding schedule, resulting in a more regulated phasic dopaminergic response that becomes associated with cues predicting access to the palatable diet [58,63]. In contrast, unpredictability keeps phasic firing elevated [10,24,64,65], consistent with our observation that across CAF days, CAFU animals exhibited a faster and greater escalation in intake than in predictable conditions (Figure 3E,G, left panels). Therefore, we suggest that both intermittency and unpredictability could potentiate behavioral sensitization. Importantly, as this framework remains largely conjectural, further research is required to elucidate how intermittency and unpredictability modulate the physiological reward response. Although the intermittent feeding schedule failed to produce significant changes in body weight, it was sufficient to promote body fat accumulation (Figure 4F) and an adverse cardiometabolic profile (Figure 5), characterized by hyperglycemia, hypertriglyceridemia, and elevated atherogenic cholesterol fractions compared to Controls, despite halved exposure to the CAF diet. These findings are consistent with those from other models using intermittent access to palatable diets [12,66].

On the other hand, all CAF-access rats showed reduced exploration of the center zone in the OFT (Figure 7B,C). These differences were not attributable to potential physical constraints induced by obesity, as they remained significant after controlling for this factor, and total locomotor activity did not differ between groups. This reduction has traditionally been interpreted as an anxiety-related behavior, suggesting that exposure to the CAF diet could also modify the emotional response to novel environments, potentially through diet-induced neuroplastic changes in the HPC—a region involved in emotional regulation and particularly sensitive to Western-style diets during early-life exposure [56,67,68]. Consistent with this interpretation, CAF diet exposure decreased DA and DOPAC levels (Figure 8) and reduced Glu concentrations in the HPC (the latter finding observed exclusively in CAFC rats), suggesting modifications in neurotransmission that could contribute to the observed anxiety-like behavior [69,70,71].

Intermittent access may further potentiate this anxiety-like phenotype, as repeated withdrawal from palatable diets has been reported to induce stress-related behaviors involving CRF1 receptor signaling in the amygdala of male Wistar rats [55,72]. We found that *Crf* mRNA levels were differentially modulated by the predictability of the intermittent access, increasing in the HPC under predictable conditions and in the mPFC under unpredictable conditions (Figure 9F). These region-specific neuroadaptations suggest that intermittent fixed access may recruit hippocampal stress-related mechanisms linked to anticipatory and contextual processing, whereas unpredictable access may preferentially engage cortical stress circuits. Given the functional connectivity between the HPC and the mPFC in regulating emotional responses, it is arguable that these adaptations may jointly contribute to the anxiety-like phenotype observed [69,73,74,75].

Consistent with previous reports [21,76,77], continuous access to the CAF diet decreased sucrose preference in the SPT (Figure 6D). This finding suggests a negative contrast in motivational and hedonic responses between the 2% sucrose solution and the highly palatable diet, such that animals likely reduced sucrose intake when a more rewarding option was anticipated. The inverse linear trend in sucrose preference (Control > CAFP > CAFU > CAFC) may reflect both the level of CAF diet exposure and reward uncertainty. We argue that this trend may involve mesolimbic dopaminergic adaptations associated with attenuated sensitivity to less salient rewards, consistent with the reduced intake of standard chow observed in all CAF-fed rats. This interpretation is also consistent with a cross-tolerance-like phenomenon, whereby extended exposure to a highly rewarding stimulus diminishes responsiveness to other rewards, similar to neuroadaptations observed in substance use disorders [41,42,47,78]. Interestingly, in the predictable intermittent group, sucrose preference was comparable to that of Controls, likely because the SPTs coincided with days when the CAF diet was not anticipated, making sucrose a relatively salient and, to some extent, unexpected reward.

Our neurochemical findings support the occurrence of diet-induced neuroadaptations in mesocorticolimbic regions. Regardless of access pattern, the CAF diet increased DOPAC levels in the mPFC (Figure 8D), indicating an elevated dopaminergic tone in the mesocortical system. We suggest that this increase could enhance the motivational value and attention to the CAF diet, which may explain the higher intake observed in CAFC rats and intermittent-access groups during CAF days. These findings are consistent with previous evidence showing that palatable diets can alter DA signaling in the PFC and impair cognitive functions, especially during adolescence [79,80]. Finally, an increase in hippocampal *Dnmt3a* mRNA levels was observed, suggesting a potential epigenetic modulation of learning and memory associated with palatable foods [81,82].

Some molecular findings appeared to depend on the pattern of access to the CAF diet. As previously reported [31], CAFC rats showed increased *Bdnf* mRNA expression in the DS (Figure 9A). This finding may reflect a transition from goal-directed to habitual food intake following continuous exposure to the CAF diet, as evidenced elsewhere [83,84]. Importantly, the increase in *Bdnf* gene expression in the DS may also be partially driven by an environmental enrichment-like effect arising from daily variations in highly palatable food items with diverse orosensory properties, such as taste, smell, and texture [27,31].

In contrast, intermittent and unpredictable access to the CAF diet increased DA turnover—a marker of elevated DA metabolism—and *Creb1* mRNA levels in the DS (Figure 8A and Figure 9C, respectively), suggesting the recruitment of the region and enhanced plasticity-related pathways involved in stimulus-response learning [85,86]. Given the role of CREB as a transcription factor, the increase in *Creb1* mRNA may reflect enhanced transcriptional activity in response to uncertainty in reward availability, possibly associated with phasic dopaminergic activity, thereby contributing to elevated intake levels during CAF days [85,87]. However, these transcriptional changes in *Creb1* mRNA were not observed at the protein level. This discrepancy could be explained by post-transcriptional regulation, temporal translation delays, post-translational modifications that affect protein stability, or compensatory mechanisms [88,89,90,91].

Conversely, compared with animals exposed to the CAF diet in an unpredictable manner, both predictable groups exhibited lower CREB and pCREB levels in the NAc (Figure 10B), with no change in the pCREB/CREB ratio. These results suggest differences in protein availability rather than in its relative activation state, which may be consistent with reduced CREB-dependent activity. Taken together, these findings suggest a potential role for striatal CREB in reward responsiveness, whereby the predictability of a highly palatable diet may modulate the recruitment of CREB-related pathways involved in motivational and hedonic processing: low predictability may be associated with greater recruitment, whereas high predictability appears to attenuate it. Overall, our results indicate that both exposure to and the pattern of access to the CAF diet could underlie region-specific adaptations across striatal circuits involved in habit formation, stimulus-response learning, and reward sensitivity [26,78].

### 4.1. Implications for Human Obesity and Health

The highest food intake observed under continuous access to the CAF diet aligns with the idea that the widespread availability of HPFs promotes overeating in humans, even in the absence of physiological hunger [92]. This could be explained by repeated activation of reward circuitry, which may override homeostatic signals, reinforce intake, and diminish the relative value of less salient but more nutritious food. In line with this, human studies have shown that individuals who attribute greater reinforcing value to food have a higher risk of future weight gain, and that repeated exposure could induce tolerance-like adaptations in reward-related circuits, such as reduced striatal responsiveness and deficits in inhibitory control, together with shifts in food preferences toward more palatable options [93,94,95,96,97,98]. The neurophysiological results presented here are consistent with these observations and provide insight into potential neurobiological substrates and mechanisms.

Under intermittent conditions, the increased food and energy intake observed during CAF days (Figure 3E–H), followed by decreased intake during chow-only days (Figure 3A–D), resembles episodic overeating in humans, such as “cheat meals,” emotional eating, weight cycling, and loss-of-control eating [99]. These behaviors are frequently observed in the context of obesity and its associated comorbidities, including type 2 diabetes, hypertension, metabolic syndrome, binge eating disorder, depression, and substance use disorders, and are important risk factors for visceral adiposity [100,101,102,103,104,105].

The anxiety-like behaviors observed in CAF-fed rats are consistent with human findings linking HPF and UPFs to a higher risk of depressive and anxiety symptoms [106]. People often engage in maladaptive coping strategies when experiencing chronic stress. While this may provide short-term relief, it can also strengthen the motivation to seek palatable foods over time [106]. In addition, periods without access to this kind of tasty food may recruit stress-related pathways, which in turn could promote emotional eating. This may be particularly relevant in the context of restrictive dieting, where such cycles can negatively affect both mental health and long-term body weight regulation [92].

Finally, the effects observed in rats under unpredictable conditions, characterized by greater escalation of food intake on CAF days, may inform understanding of human eating behaviors when food access becomes unpredictable. In humans, uncertainty in food access can arise from irregular meal schedules, restrictive eating patterns, constant exposure to food cues, and stress. In addition, people living with food insecurity face uncertain access to adequate foods [107]. Despite limited access to food, these individuals tend to have higher BMI, increased abdominal adiposity, poorer diet quality, and a higher risk of metabolic diseases [108]. These associations have been linked to motivational processes that encourage greater consumption of low-cost, ultra-processed, and HPFs, while the stress associated with food insecurity may further exacerbate emotional eating [24,108].

### 4.2. Limitations

This study has some limitations. First, the exclusive inclusion of male rats does not account for sex-dependent differences in metabolic, behavioral, and neuroadaptive responses in reward-related brain regions. Although the CAF diet has ecological validity, it does not fully capture the complex biological and environmental factors that influence food intake behaviors in humans. The behavioral assessment was limited to two tests; additional tests could have assessed more specific anxiety-like behaviors and other dimensions that distinguish motivational from hedonic-driven responses. Finally, molecular measurements were conducted at a single endpoint, preventing assessment of the temporal progression of behavioral and neurobiological changes.

## 5. Conclusions

Taken together, these findings underscore that not only diet composition but also patterns and predictability of food intake play a key role in feeding behavior. The greater escalation in intake observed under unpredictable conditions during CAF days highlights that uncertainty enhances the motivational value of food, thereby promoting hyperphagia. Intermittent access to the CAF diet was sufficient to increase body fat and induce adverse metabolic changes despite the lack of significant differences in final body weight relative to Controls.

Moreover, exposure to the CAF diet and the access pattern led to specific neuroadaptations in brain regions involved in reward processing, habit formation, memory, decision-making, and emotional regulation. These changes may underlie reward-driven behaviors, behavioral sensitization, cross-tolerance-like phenomena, and involvement of stress-related pathways. This is supported by the escalation of intake on CAF days under intermittent and unpredictable conditions, reduced consumption of less palatable rewards (i.e., chow pellets and 2% sucrose solution), and stress-related behaviors in all CAF-fed rats, as well as altered *Crf* mRNA levels in key brain regions involved in emotional regulation, depending on predictability.

Our results highlight potential mechanisms through which the pattern of HPF availability drives eating behavior, with implications for metabolic and mental health outcomes. Further research should include female rats to account for sex-dependent differences in feeding and stress-related behaviors, as well as in brain reward-related regions following exposure to highly palatable diets. To better understand the neurochemical and molecular changes we observed, follow-up studies with temporal and circuit-level analyses, epigenetic approaches, measures of hypothalamic–pituitary–adrenal axis activity, and investigation of the persistence or reversibility of these effects are warranted.

Overall, our findings highlight the urgent need for public health policies targeting the modern food environment, including the intense marketing of UPFs to adolescents, as exposure to HPFs during critical periods of brain development could lead to lasting neuroadaptations that affect cognitive function, mental health, and the risk of obesity-related non-communicable diseases in adulthood.

## Data Availability

The original contributions presented in this study are included in the article and Supplementary Materials. Further inquiries can be directed to the corresponding author.

## Supplementary Materials

The following supporting information can be downloaded at: https://www.mdpi.com/article/10.3390/nu18121913/s1, Table S1: Nutritional composition of the cafeteria diet food items provided by the manufacturer; Table S2: Frequency of food item inclusion across cafeteria diet combinations during the experimental protocol; Statistical analysis; Figure S1: Overall mean food and energy intake across the whole experiment (A–D), chow-only days (E–H), and CAF days (I–L); Table S3: Ex vivo concentration of neurotransmitters in rats exposed to different patterns.

## Abbreviations

The following abbreviations are used in this manuscript:

HPF	Highly Palatable Food
UPF	Ultra-processed Food
CAF	Cafeteria diet
NAc	Nucleus accumbens
DS	Dorsal striatum
mPFC	Medial prefrontal cortex
HPC	Hippocampus
BMI	Body mass index
SPT	Sucrose preference test
OFT	Open field test
TC	Total cholesterol
HDL	High-density lipoprotein cholesterol
TG	Triglycerides
HPLC	High-performance liquid chromatography
DA	Dopamine
DOPAC	3,4-dihydroxyphenylacetic acid
HVA	Homovanillic acid
5-HT	5-hydroxytryptamine
5-HIAA	5-hydroxyindoleacetic acid
NE	Norepinephrine
Glu	Glutamate
Gln	Glutamine
GABA	Gamma-aminobutyric acid
Bdnf	Brain-derived neurotrophic factor
Ntrk2	Neurotrophic receptor tyrosine kinase 2
Dnmt3a	DNA methyltransferase 3A
Hdac5	Histone deacetylase 5
Crf	Corticotropin-releasing factor
Creb1	cAMP response element-binding protein 1
