# Supplementary material for: Intermittency and Predictability of a Cafeteria Diet Shape Food Intake, Adiposity, and Neurobehavioral Outcomes in Rats
Source: Nutrients. 2026 Jun 12;18(12):1913. doi: 10.3390/nu18121913 (PMC13305956; doi:10.3390/nu18121913)
Supplement: Supplementary file 1 [file nutrients-18-01913-s001.zip › nutrients-4322961-supplementary.pdf]

Supplementary Materials

Supplementary Materials and Methods

Experimental design

Table S1. Nutritional composition of the cafeteria diet food items provided by the manufacturer.

Food[a]	kcal	Carbohydrates	Fat	Protein	Fiber	Sugar
Standard chow	3.41	0.50	0.05	0.24	0.04	0.01
Corn and cheese snack	4.97	0.60	0.24	0.07	0.01	0.00
Wafers	4.89	0.79	0.18	0.04	0.02	0.32
Jelly filled cookies	4.22	0.62	0.18	0.04	0.09	0.22
Coconut cookies	4.81	0.65	0.23	0.08	0.01	0.19
Fruit flavored cereal	4.06	0.88	0.03	0.06	0.03	0.47
Hot dog bread	2.90	0.48	0.07	0.10	0.02	0.06
Hot dogs	2.32	0.05	0.19	0.11	0.00	0.00
Chocolate-cream sandwich cookie	4.72	0.72	0.19	0.06	0.02	0.44
Salty peanut	5.71	0.18	0.50	0.25	0.07	0.00
Cheese puffs	5.67	0.50	0.37	0.07	0.07	0.00
Sausage	2.28	0.10	0.16	0.11	0.00	0.00
Cheese	3.33	0.03	0.27	0.20	0.00	0.00
Coconut flavored-cream sandwich cookie	5.08	0.68	0.25	0.03	0.02	0.32
Chocolate covered wafer	5.14	0.64	0.28	0.04	0.03	0.53
Honey flavored ring cereal	3.93	0.79	0.05	0.07	0.07	0.32
Crunchy butter cookies	4.77	0.49	0.25	0.07	0.03	0.29

[a]Values correspond to 1 g of food. All macronutrients are expressed in grams.

Table S2. Frequency of food item inclusion across cafeteria diet combinations during the experimental protocol.

Food item	CAFP	CAFU	CAFC
	Frequency (*n*)		
Standard chow	31	31	68
Corn and cheese snack	6	6	14
Wafers	6	6	11
Jelly filled cookies	6	7	16
Coconut cookies	8	6	14
Fruit flavored cereal	4	5	11
Hot dog bread	5	6	11
Hot dogs	5	9	19
Chocolate-cream sandwich cookie	6	4	10
Salty peanut	7	3	10
Cheese puffs	8	6	17
Sausage	3	5	12
Cheese	8	9	18
Coconut-cream sandwich cookie	5	7	10
Chocolate covered wafer	9	4	12
Honey flavored ring cereal	3	4	8
Crunchy butter cookies	4	6	11

CAFP: cafeteria predictable intermittent-access group; CAFU: cafeteria unpredictable intermittent-access group; CAFC: cafeteria continuous-access group.

Statistical analysis

Weekly mean daily food intake and body weight per animal were analyzed using a one-way mixed multivariate analysis of covariance (MANCOVA), with Week as the within-subject factor and Access Pattern to the CAF diet (Control, CAFP, CAFU, and CAFC) as the between-subject factor. The dam was included as a covariate in all analyses to account for genetic or maternal care effects, as previously described [1]. Locomotor activity in the OFT was analyzed using mixed-design models. Total distance traveled, distance and time spent in the center, and the frequency and duration of rearing and grooming behaviors across the two OFT sessions were analyzed using mixed ANCOVA, with Test (OFT 1 and OFT 2) as the within-subject factor and Access Pattern as the between-subject factor. Similarly, water and sucrose intake and sucrose preference were analyzed using a mixed ANCOVA, with Test (SPT 1 and SPT 2) as the within-subject factor and Access Pattern as the between-subject factor. When the assumption of sphericity was violated (Mauchly's test), Greenhouse–Geisser corrections were applied. Overall mean weekly food intake and body weight, together with mean biometric parameters, serum biochemical variables, behavioral outcomes, neurochemical, and molecular measures (i.e., mRNA and protein levels) were

analyzed using one-way ANCOVA, with Access Pattern as the between-subject factor. When appropriate, pairwise comparisons were conducted using planned contrasts to test *a priori* hypotheses or based on graphical inspection of the data. Reverse Helmert contrasts were used for comparisons of CAFP vs CAFU, CAFC vs. intermittent CAF groups, and Control vs. all CAF groups, while simple contrasts were applied when a specific group differed from the others. If a main effect of Diet (chow vs. CAF) was suggested, Control vs. all CAF groups was tested. If a main effect of Predictability was observed, predictable access groups (CAFP and CAFC) were compared against CAFU. Data are expressed as estimated marginal means + standard error of the mean (SEM) derived from the ANCOVA models. Partial eta squared (η^2_p) was reported for significant main effects of Access Pattern or Diet, and Cohen's *d* was used for pairwise comparisons. Statistical analyses were performed by an investigator aware of group allocation, using uniform software (IBM® SPSS Statistics software (v25.0; IBM Corp., Armonk, NY, USA) and criteria across groups to minimize bias. Statistical significance was set at $p \leq 0.05$. Graphical representations were generated using GraphPad Prism (v10.6.1; GraphPad Software, San Diego, CA, USA).

Supplementary Results

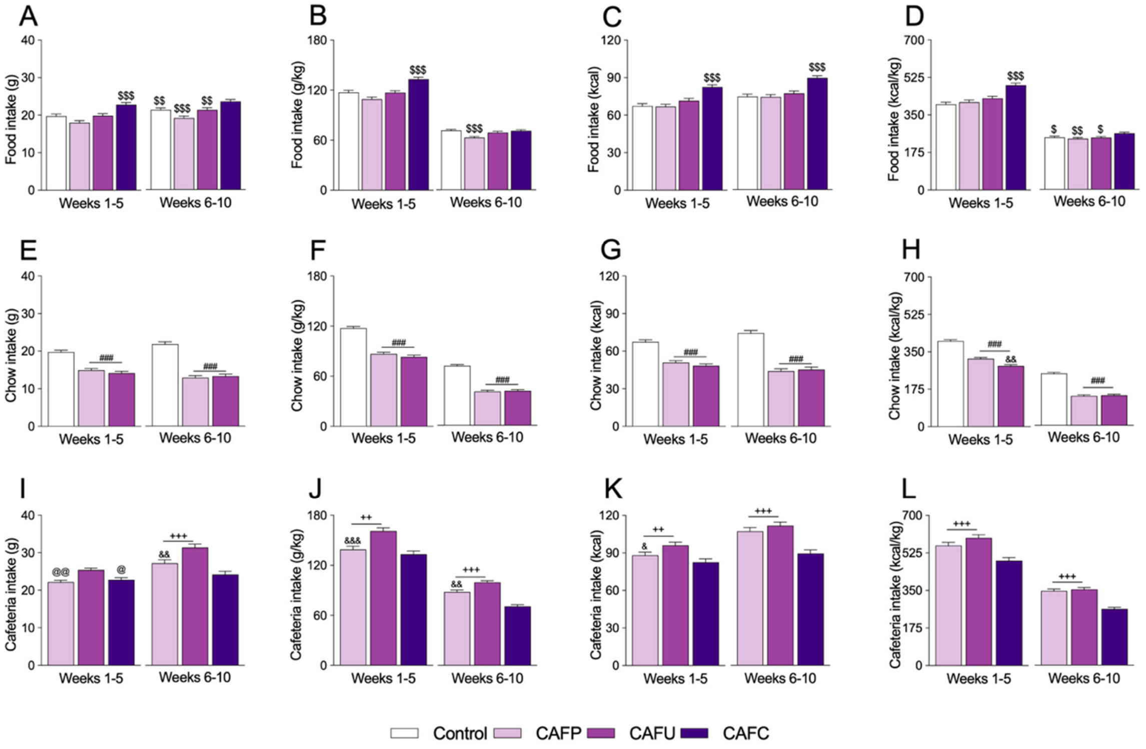

Figure S1. Overall mean food and energy intake across the whole experiment (A–D), chow-only days (E–H), and CAF days (I–L). (A) Total food intake. (B) Total food intake normalized to body weight (g/kg). (C) Total food energy intake (D) Total food energy intake normalized to body weight (kcal/kg). (E) Chow intake. (F) Chow intake normalized to body weight (g/kg). (G) Chow-derived energy intake. (H) Chow-derived energy intake normalized to body weight (kcal/kg). (I) CAF diet intake. (J) CAF diet-derived energy intake normalized to body weight (g/kg). (K) CAF diet-derived energy intake. (L) CAF diet-derived energy intake normalized to body weight (kcal/kg). All left panels correspond to weeks 1–5, and right panels to weeks 6–10. Data are presented as estimated marginal means + SEM from ANCOVAs (*n* = 10 animals/ group). Symbols denote planned contrasts: CAFC vs. other groups: \$ $p < 0.05$, \$\$ $p < 0.01$, \$\$\$ $p < 0.001$; Control group vs. intermittent-access groups: ### $p < 0.001$; CAFC vs. intermittent-access groups: ++ $p < 0.01$, +++ $p < 0.001$; CAFP vs. CAFU: & $p < 0.05$, && $p < 0.01$, &&& $p < 0.001$; CAFU vs. CAFP and CAFC groups: @ $p < 0.05$, @@ $p < 0.01$. Exclusively chow-fed rats constitute the Control group. CAFP: cafeteria predictable intermittent-access group; CAFU: cafeteria unpredictable intermittent-access group; CAFC: cafeteria continuous-access group.

Table S3. Ex-vivo concentration of neurotransmitters in rats exposed to different patterns

Neurotransmitter	Brain area	Control	CAFP	CAFU	CAFC
NE	DS	0.166±0.012	0.168±0.021	0.164±0.014	0.187±0.016
	NAc	0.621±0.082	0.674±0.136	0.495 ±0.079	0.507 ±0.087
	mPFC	0.300±0.014	0.302±0.016	0.322±0.013	0.319±0.024
	HPC	0.320±0.019	0.312±0.013	0.324±0.017	0.292±0.018
5-HT	DS	0.599±0.030	0.599±0.042	0.550±0.045	0.669±0.050
	NAc	2.019±0.281	1.798±0.151	1.820±0.286	1.573±0.140
	mPFC	0.576±0.018	0.546±0.036	0.600±0.048	0.522±0.046
	HPC	0.340±0.019	0.325±0.019	0.363±0.013	0.306±0.021
5-HIAA	DS	0.744±0.034	0.748±0.050	0.758±0.041	0.825±0.037
	NAc	1.218±0.092	1.308±0.122	1.793±0.497	1.086±0.161
	mPFC	0.338±0.026	0.310±0.026	0.381±0.051	0.428±0.035
	HPC	0.291±0.024	0.244±0.020	0.268±0.016	0.286±0.046
5-HT turnover	DS	1.256±0.056	1.280±0.092	1.435±0.098	1.275±0.085
	NAc	0.661±0.067	0.740±0.051	0.963±0.212	0.673±0.050
	mPFC	0.585±0.037	0.577±0.042	0.714±0.157	0.941±0.202
	HPC	0.873±0.081	0.748±0.050	0.743±0.042	0.982±0.198
Glu	DS	1.005±0.038	0.962±0.062	1.066±0.061	1.242±0.166
	NAc	1.477±0.238	1.582±0.236	1.236±0.132	1.088±0.144
	mPFC	1.478±0.082	1.464±0.079	1.634±0.071	1.571±0.098
	HPC	**1.163±0.089**	1.039±0.053	1.023±0.047	**0.844±0.081**$
Gln	DS	2.624±0.208	3.172±0.576	2.405±0.147	3.702±0.553
	NAc	3.230±0.628	3.138±0.379	2.639±0.412	2.182±0.371
	mPFC	4.057±0.152	4.086±0.780	4.249±0.821	4.443±0.492
	HPC	1.831±0.285	1.365±0.207	1.436±0.183	1.086±0.149
GABA	DS	0.181±0.006	0.144±0.011	0.175±0.008	0.216±0.036
	NAc	0.478±0.052	0.448±0.079	0.414±0.046	0.339±0.037
	mPFC	0.152±0.015	0.149±0.016	0.152±0.018	1.171±0.011
	HPC	0.177±0.014	0.150±0.008	0.160±0.008	0.137±0.015

Data are mean ± SEM (n = 10 animals per group) and were estimated from MANCOVA analysis. Estimated mean values correspond to ng/mg of tissue. NE: norepinephrine; 5-HT: 5-hydroxytryptamine; 5-HIAA: 5-hydroxyindoleacetic acid; 5-HT turnover: 5-HIAA/5-HT; Glu: glutamate; Gln: glutamine; GABA: gamma-aminobutyric acid; DS: dorsal striatum; NAc: nucleus accumbens; mPFC: medial prefrontal cortex; HPC: hippocampus. Bold numbers indicate significant values. Symbols denote planned contrasts: CAFC vs. Control group: $ $p < 0.05$. Exclusively chow-fed rats constitute the Control group. CAFP: cafeteria predictable intermittent-access group; CAFU: cafeteria unpredictable intermittent-access group; CAFC: cafeteria continuous-access group.

Supplementary References

1. Vindas-Smith, R.; Quesada, D.; Hernández-Solano, M.I.; Castro, M.; Sequeira-Cordero, A.; Fornaguera, J.; Gómez, G.; Brenes, J.C. Fat Intake and Obesity-Related Parameters Predict Striatal BDNF Gene Expression and Dopamine Metabolite Levels in Cafeteria Diet-Fed Rats. *Neuroscience* **2022**, *491*, 225–239, doi:10.1016/j.neuroscience.2022.03.042.
